# Green vehicle routing optimization based on dynamic constraint selection co-evolutionary algorithm

**DOI:** 10.1038/s41598-025-01480-7

**Published:** 2025-05-21

**Authors:** Lu-jie Zhou, Hai-fei Zhang, Jun-hao Fu

**Affiliations:** 1 School of Automobile and Transportation, Tianjin University of Technology and Education, Tianjin, 300222 China; 2 Nanjing Xiaozhuang University, College of information engineering, Nanjing, China; 3 School of Artificial Intelligence and Computer Science, Jiangnan University, Wuxi, 214122 Jiangsu China; 4 Key Laboratory of Advanced Process Control for Light Industry (Jiangnan University), Ministry of Education, Wuxi, 214122 China

**Keywords:** Logistics distribution, Multi-objective optimization, Time-varying property, Co-evolutionary algorithm, Dynamic constraint selection, Engineering, Mathematics and computing

## Abstract

Aiming at the problems of single solution objective in the existing green vehicle routing optimization process and real-time speed change during vehicle travel, a multi-objective green vehicle routing problem with time window constraints in time-varying conditions(MOGVRPTW-TV) is established, then a co-evolutionary framework-based constrained multi-objective evolutionary algorithm for the solving of the model is proposed. First, to better match the actual logistics distribution, this model considers the impact of time-varying speed and capacitated variation on carbon emissions based on capacitated constraints and time window constraints. Second, a constrained multi-objective evolutionary algorithm based on the co-evolutionary framework was proposed for model solving. The algorithm treats the complete problem model as a complex task and introduces a shift crowding distance calculation that considers both individual distribution and convergence information when solving this complex task, effectively balancing the convergence and diversity of solutions. Then, a dynamic constraint selection strategy is designed for the implementation of the simple task, which only considers the effects of some constraints on the populations, and the two populations exchange information through offspring populations to achieve overall optimization. Simulations are performed on different instances and the results show that the proposed algorithm is effective in avoiding traffic congestion periods, decreasing overall distribution costs, and reducing fuel consumption and carbon emissions of vehicles.

## Introduction

With the improvement of the logistics infrastructure layout, the logistics industry has become a key link in the development of the national economy, and vehicle transportation occupies an important position in the entire logistics service process^1^. Efficient routing planning solutions can reduce vehicle transportation costs, improve the efficiency of distribution centers, and improve service quality while meeting a wide variety of customer requirements. With the gradual enhancement of customers’ time concept, the vehicle routing problem with time window (VRPTW) has become a research hotspot. Faced with the problem of energy consumption, it is urgent to conduct research on low-carbon vehicle routing problem (VRP) to promote energy saving and emission reduction^2^. Therefore, MOGVRPTW-TV is investigated in this paper to make a reasonable and efficient routing planning for the distribution tasks while satisfying the customer’s requirements on the distribution service time and minimizing the carbon emissions.

The main method for solving vehicle routing problems is to establish mathematical models, define different types of variables, constraint functions, and objective functions to complete optimization of the vehicle routing. There are three main approaches^3–6^: the first is the accurate search algorithm, which establishes a corresponding mathematical model for a particular problem and uses mathematical methods to find optimal solutions^7–10^. Due to VRP being an NP hard problem, accurate algorithms rely on the solution space, number of constraints, and number of decision variables, making it difficult to offer universal solutions. This will consume more computing power and storage space, making it suitable for small scale VRP solving. As the scale of the problem gradually increases, partial literatures proposed using heuristic algorithms to solve VRP. The basic idea of heuristic algorithms is to design heuristic functions to provide a feasible solution to the problem to be solved within an acceptable range^11–16^. Compared to the former, when handling large scale VRPs, the heuristic algorithms showcased better feasibility, and can search for the optimal solution of the problem in a short time, which improved vehicle routing’s accuracy further.

For VRPTW it is important to both minimize the cost by choosing reasonable routes in the geographical space and to arrange an efficient customer service sequence based on the customer’s service time at the time level. VRPTW can be categorized into hard and soft time window^17^.The hard time windows require vehicles to provide service at the start of the time window, while the soft time window replaces restrictions such as waiting and rejecting with penalties. The current research methods for solving VRPTW and related variants are shown in Table1.

**Table 1:** VRPTW solution methods.

References	Problem	Method
Yuan et al.^18^	VRPTW	A hybrid heuristic algorithm for service time customization based on multi-ant system
Iqbal et al.^19^	VRPTW	Hybrid hyper-heuristic algorithm
Cheng et al.^20^ Zhang^21^	VRPTW	Multi-task optimization framework combined with multi-objective evolutionary algorithms
Jin et al.^22^	VRPTW	Hybrid algorithm combining tabu search and artificial immunity algorithms
Melián-Batista et al.^23^	Dual-objective VRPTW	A meta-heuristic algorithm based on scatter search
Shu et al.^24^	Triple-objective VRPTW	A two-stage algorithm that classifies populations in the first stage and performs a multi-objective search in the second stage
Ghoseiri et al.^25^	Multi-objective VRPTW	A multi-objective genetic algorithm
Alvarenga^26^	Multi-objective VRPTW	Two stage optimization, first the number of vehicles and then the cost
Tian^27^	Multi-objective VRPTW	Co-evolutionary framework based on NSGA-II
Qi^28^	Multi-objective VRPTW	Decomposition-based modal factorization algorithms
Hou et al.^29^	Multi-objective VRPTW	Multi-objective differential evolutionary algorithms for solution-oriented evaluation

With the substantial growth of logistics and transport activities, the direct or indirect harmful effects of transportation on humans and ecosystems are becoming increasingly severe. Since Erdoğan and Miller-Hooks^30^ introduced GVRP as an extension of VRP in research, the main focus of GVRP has been on the impact of vehicle speed, load capacity, and vehicle type on carbon emissions during transportation. The research methods for solving GVRP are shown in Table 2.

**Table 2:** GVRP solution methods.

References	Problem	Method
Demir et al.^31^	GVRP	Adaptive large neighborhood search heuristic algorithm
Poonthalir et al.^32^	GVRP with variable speed constraints	Particle swarm optimization algorithm using particle swarm with time-varying acceleration coefficients
Kunnapapdeelert et al.^33^	GVRP with time window	Differential evolutionary algorithm that calculates carbon emissions based on distance traveled, without considering other factors such as speed and weight of load
Niu et al.^34^	GVRP with randomized demand	Membrane inspired multi-objective algorithm

Based on the above literature analysis, there are still the following issues in the current research on VRP and its variants: (1) At present, research on green vehicle routing problems mainly focuses on the integration of carbon emissions and time window constraints, while there is relatively little research on multi-objective vehicle routing problems that comprehensively consider the driving cost, fixed cost, and carbon emission cost caused by speed and load in real demand. (2) For the multi-objective function of VRP, a weighted sum method is usually used for solving, and the weight coefficients are often set based on experience, resulting in less accurate solution results. (3) When solving the model, the quality of the algorithm’s solution decreases significantly as the number of objectives increases, while the number of non-inferior solutions required by Pareto increases exponentially, making it difficult to solve for better routes. (4) The consideration of the real conditions is relatively single, which cannot well reflect the requirements of the actual logistics and distribution process on the route planning.

Based on the above analysis, this paper proposes a MOGVRPTW-TV model on the basis of previous studies, and designs a constrained multi-objective evolutionary algorithm based on the co-evolutionary framework for solving the solving of the model.

The main contributions of this paper can be summarized below:

1. To improve logistics distribution efficiency and achieve more realistic logistics distribution needs, a multi-objective green vehicle routing problem model with time window constraints is established in a time-varying environment, taking into account driving costs, fixed costs, and carbon emission costs.
2. Based on the idea of simple task assisting complex task solution in multi-task optimization, a constrained multi-objective evolutionary algorithm based on co-evolutionary framework is proposed for solving the MOGVRPTW-TV model. The algorithm takes the complete MOGVRPTW-TV as a complex task, and introduces a shifted congestion distance computation that takes into account both individual distribution and convergence information when solving the complex task as a way of balancing the convergence and diversity of the solution.
3. A dynamic constraint selection strategy is designed for the realization of a simple task, which only considers the effect of some constraints on the populations, and the two populations exchange information through offspring populations.
4. The efficiency of the proposed algorithm is evaluated on several benchmark datasets and actual VRPTW instances to validate the performance of the proposed algorithm.

## Problem description and model building

### Problem description

In actual logistics and distribution, the speed of vehicles is related to departure time and road conditions, and the carbon emissions of vehicles also affect the development of the city.

Therefore, this paper proposes a multi-objective vehicle routing problem model with time window constraints, considering the total transportation cost, total travel time, and number of vehicles of carbon emissions in a time-varying environment (MOGVRPTW-TV), where the carbon emissions comprehensively consider factors such as speed, load capacity, and distance.

The MOGVRPTW-TV is described as: in a distribution region, there is a distribution center at a known location and several vehicles sent from the distribution center to complete the logistics and distribution of different customers in the distribution region in an orderly and non-repetitive manner. Each vehicle has a maximum capacity constraint and each customer node has a distribution soft time window constraint, where the speed of the vehicle varies due to road congestion in different time periods. Under the above constraints, the vehicle routings are rationally planned to ultimately achieve the optimal goals of the total logistics distribution cost, the total travel time and number of dispatched vehicles.

Due to the complexity and variability of the actual situation, the following assumptions are made in the modeling of the VRP with time window constraint to better conduct study.

1. There is only one distribution center and the location is known.
2. Each customer must and can only be visited once, and the demands cannot be split.
3. The time window that the customer wants to be visited is known, and the delay time must not exceed the maximum allowable delay time. If the vehicle arrives earlier than the time window, it is necessary to wait.
4. The weight of the customer’s goods is equal to the demand.
5. Vehicles are limited in carrying capacity and have no maximum transportation travel limitations.
6. All vehicles have the same capacity and driving speed, and have a maximum distance limit.
7. The vehicles must start from the distribution center and finally return to the distribution center.
8. Vehicle travels without considering the effects of slope.
9. No pauses during vehicle travel, such as waiting for traffic lights.

## Notation definition

The notations and meanings related to the model are represented in Table 3.

**Table 3:** Notation definition.

Notation	Meaning	Notation	Meaning
$V = \{ 0...,N\}$	The set of vertices	$\{ 1,...,n\}$	The customer number
$A$	The set of arcs	$Cap$	Capacity of vehicles
*WV*	The weight of the vehicle	$N_{j}$	The number of customers served by vehicle $j$
$CE$	The formula for calculating carbon emissions	$c_{t}$	The penalty cost per unit delay time
$delay_{h(i,j)}$	The delay time of vehicle $j$ at the *i*-th node in the route	$h(i,j)$	The customer number of the *i*-th node in the traveling route of vehicle $j$
$c_{d}$	The travel cost per unit distance	$c_{v}$	Vehicle fixed costs
$T_{j}$	The travel time of vehicle $j$	$x_{ijk}$	The decision variable
$weight_{h(i,k)}$	the weight of vehicle $k$ when it travels to the *i*-th node in the route	$\alpha$	The carbon emission conversion factor
$v$	The speed of vehicles	$g$	The vehicle load capacity
$d$	The distance traveled by a single vehicle	$l$	The time period

### MOGVRPTW-TV model construction

Consider MOGVRPTW-TV as a graph $G = (V,A)$, where $V = \{ 0...,N\}$ is the set of vertices, vertex 0 is the distribution center, $\{ 1,...,n\}$ is the customer number, and $A$ is the set of arcs. Distribution centers use a fleet of the same type of transport vehicles with capacity $Cap$ to serve these customers. Each customer has a requirement $m_{i}$ and a time window $[b_{i} ,e_{i} ]$. Each customer $i$ has a distribution time $s_{i}$. $s_{i}$ is the time it takes for each vehicle to deliver one shipment after it arrives at the customer node and starts the distribution task. The time window for the distribution center is $[0,e_{0} ]$. The distance between any two customer nodes $i$ and $j$ is $d_{ij}$. The travel time is $t_{ij}$. The goal of this problem is to find the optimal solution of the objective function as much as possible for a set of routings $R = \{ r_{1} ,...,r_{M} \}$ while satisfying constraints.

Based on the above assumptions and definitions, the following mixed integer programming mathematical model is established.

The three objectives considered by the model are:

1 $$\min f = \{ f_{1} ,f_{2} ,f_{3} \}$$

The total cost of all vehicles is $f_{1}$, which includes carbon emission costs, time delay costs, vehicle fixed costs, and travel costs:

The total transportation time of all vehicles during distribution includes the travel time and waiting time of vehicles:

2 $$f_{1} = \sum\limits_{j = 1}^{M} {\sum\limits_{i = 0}^{{N_{j} }} {\left( {CE + c_{t} delay_{h(i,j)} + c_{d} d_{h(i,j)h(i + 1,j)} } \right) + c_{v} M} }$$

3 $$f_{2} = \sum\limits_{j = 1}^{M} {T_{j} }$$

The number of vehicles needed to complete this distribution task is:

4 $$f_{3} = |R| = M$$

While travel distance and time are correlated (e.g., under constant speed), their coupling is nonlinear under time-varying speeds. Our model accounts for time-varying traffic conditions, where the shortest path (minimizing distance) may not always align with the fastest path (minimizing time). In addition, fixed costs are only related to whether vehicles are assigned tasks (that is, used), rather than simply the number of vehicles. In addition, the number of vehicles is independent of fixed costs and is reflected by other costs such as parking and management.

The model contains various constraints, such as capacity constraints, time window constraints. The specific definitions of the constraints is as follows:

Each customer is guaranteed to be serviced by only one vehicle:

5 $$\sum\limits_{k = 1}^{M} {\sum\limits_{j \in V}^{{}} {x_{ijk} } } = 1,\;i \in V\backslash \{ 0\}$$

where, $x_{ijk}$ is the decision variable, if vehicle $k$ passes through arc $(i,j)$, it is 1, otherwise it is 0.

Each vehicle can complete a distribution task at most once:

6 $$\sum\limits_{i \in V} {x_{0jk} \le 1,\;\forall \;k \in \{ 1,...,M\} }$$

After each vehicle enters a customer node, it must still drive out of that customer node:

7 $$\sum\limits_{i \in V} {x_{ijk} } - \sum\limits_{j \in V} {x_{jik} } = 0,\;\forall \,k \in \{ 1,...,M\}$$

The vehicle must exit the distribution center and return to the distribution center:

8 $$\sum\limits_{j \in V} {x_{0jk} } = \sum\limits_{i \in V} {x_{i0k} } ,\,\forall \;k \in \{ 1,...,M\}$$

Vehicles must not be loaded with more than the loading capacity:

9 $$weight_{h(1,k)} \le Cap,\;\forall \,k \in \{ 1,...,M\}$$

All vehicles should return to the distribution center before the end of the time window:

10 $$a_{{h(N_{i + 1} ,k)}} \le e_{0} ,\;\forall \,i \in V,\,\,k \in \{ 1,...,M\}$$

The delay time of vehicles on all routings cannot exceed the maximum allowable delay time:

11 $$delay_{h(i,k)} \le md,\forall i \in \{ 1,...,N_{j} \} ,\forall k \in \{ 1,...,M\}$$

The speed of vehicles is related to the departure time, so the travel time of each routing needs to be dynamically calculated. The calculation of carbon emissions is related to the vehicle’s capacity. Therefore, under the dynamic conditions, the weight of the vehicles and the travel time of the routings are calculated as follows:

For the convenience of calculation, the starting and ending points of each route are set as the distribution center.

12 $$h(0,k) = h(N_{j} + 1,k) = 0$$

The waiting time $w_{h(i,k)}$ is calculated as follows:

13 $$w_{h(i,k)} = \left\{ {\begin{array}{*{20}l} {0,} \hfill & {if{\kern 1pt} a_{h(i,k)} \ge b_{h(i,k)} - md} \hfill \\ {b_{h(i,k)} - a_{h(i,k)} ,} \hfill & {otherwise} \hfill \\ \end{array} } \right.$$

The departure time $l_{h(i,k)}$ is calculated as follows:

14 $$l_{h(i,k)} = a_{h(i,k)} + w_{h(i,k)} + s_{h(i,k)}$$

The arrival time $a_{h(i,k)}$ is calculated as follows:

15 $$a_{h(i,k)} = l_{h(i - 1,k)} + t_{h(i - 1,k)h(i,k)}$$

where, $t_{h(i - 1,k)h(i,k)}$ denotes the travel time of vehicle $k$ from node $i - 1$-th to node $i$-th in the route, which is calculated based on distance, speed, and departure time. The calculation is as follows:

16 $$t_{h(i - 1,k)h(i,k)} = ride(l_{h(i - 1,k)} ,d_{h(i - 1,k)h(i,k)} )$$

The delay time $delay_{h(i,k)}$ is calculated as follows:

17 $$delay_{h(i,k)} = \left\{ {\begin{array}{*{20}l} {0,} \hfill & {{\kern 1pt} if{\kern 1pt} {\kern 1pt} a_{h(i,k)} \le e_{h(i,k)} } \hfill \\ {a_{h(i,k)} - e_{h(i,k)} ,} \hfill & {otherwise} \hfill \\ \end{array} } \right.$$

Total travel time of vehicles on each routing:

18 $$T_{k} = \sum\limits_{i = 0}^{{N_{j} }} {\left( {t_{h(i,k)h(i + 1,k)} + w_{h(i,k)} + s_{h(i,k)} } \right)}$$

The formula for the total weight change of each vehicle is as follows:

19 $$weight_{h(i,k)} = weight_{h(i - 1,k)} - m_{h(i - 1,k)}$$

Carbon emission formula $CE$ is calculated as follows:

20 $$CE(v,g,d,l) = \alpha * FC(v,g,d,l) = \alpha * \frac{g \times d}{{v \times l}}$$

In the time-varying model adopted in this paper, the delivery time is divided into $L$ segments. The speed of each segment may be different, and the vehicle has a great probability to span multiple time segments during the driving process. The travel time calculation function $ride(t_{begin} ,d)$ between customer $i$ and customer $j$ is shown in Algorithm 1.

**Algorithm 1:**
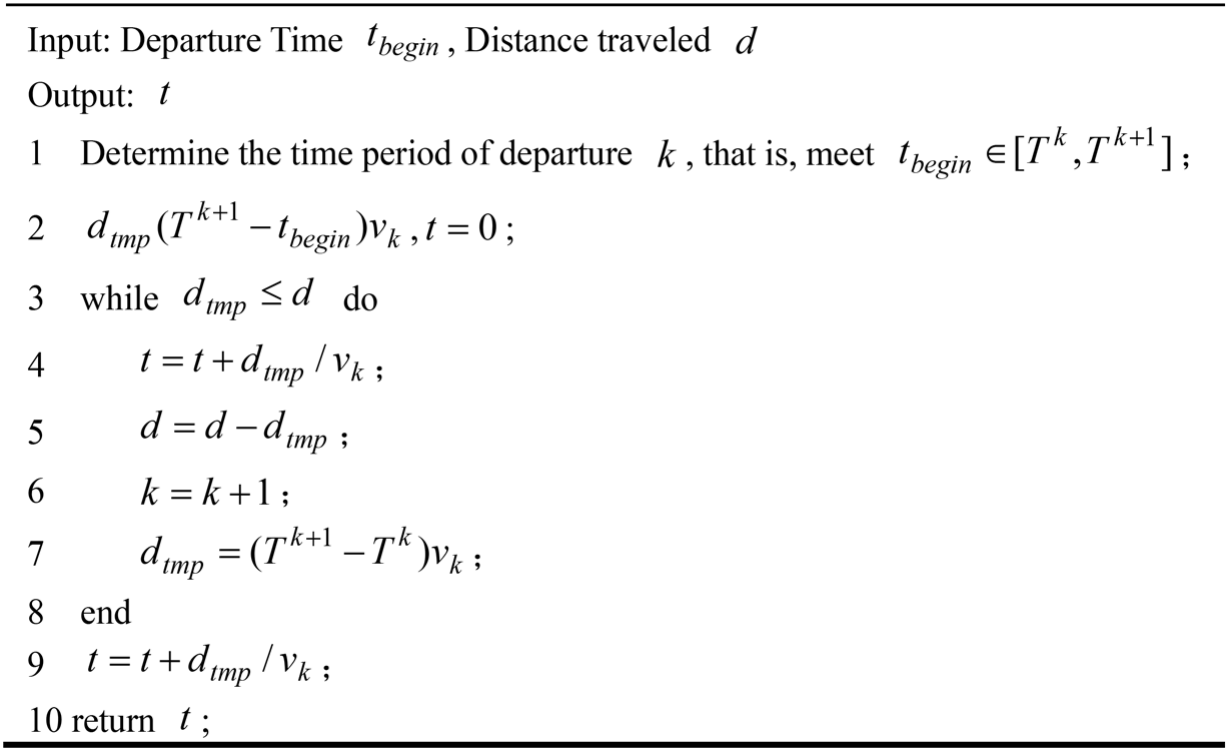
$$ride(t_{begin} ,d)$$

## Constrained multi-objective evolutionary algorithm based on cooperative co-evolutionary framework

### Algorithm framework

**Fig. 1:**
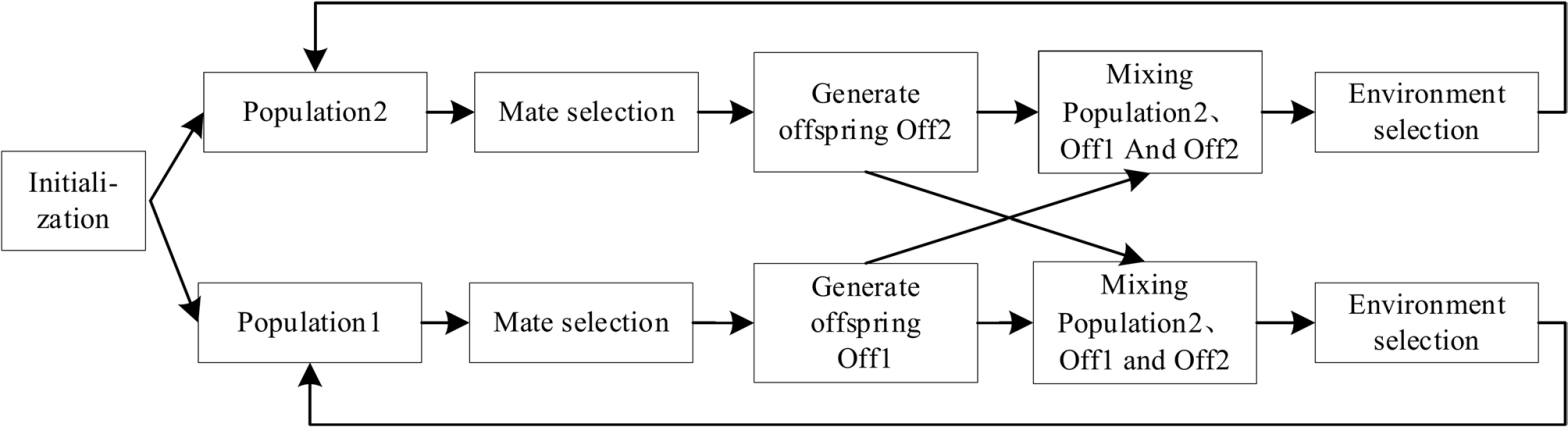
Framework of co-evolutionary algorithm.

To better balance convergence, diversity, and feasibility, this chapter proposes a Coevolution Dynamic Select Constrained Multiobjective Optimization (CDSMO) algorithm based on the cooperative co-evolutionary framework^35^. The algorithm maintains two subpopulations simultaneously, namely $Population{\kern 1pt} {\kern 1pt} {\kern 1pt} {\kern 1pt} {\kern 1pt} 1$ and $Population{\kern 1pt} {\kern 1pt} {\kern 1pt} {\kern 1pt} 2$. According to the idea of simple tasks assisting complex tasks in multi-task optimization, the main population $Population{\kern 1pt} {\kern 1pt} {\kern 1pt} {\kern 1pt} {\kern 1pt} 1$ is used to solve the complete and complex original problem, and the auxiliary population $Population{\kern 1pt} {\kern 1pt} {\kern 1pt} {\kern 1pt} 2$ dynamically balances the impact of the constraints on the solution of the original problem, making the task of reducing constraint constraints a simple task. For the auxiliary population, less constraint information is considered and convergence is faster compared to the main population. The information exchange between two populations can accelerate the convergence speed of the main population. The main population takes into account the complete constraint information, and the information exchange can better guide the search of the auxiliary population. For vehicle route optimization with time window constraints, a complex task is a vehicle route optimization that satisfies all the constraints, while a simple task is a vehicle route optimization that can violate some of the constraints such as time window and capacity. The main framework of the co-evolutionary algorithm is shown in Fig. 1.

The two populations of the CDSMO algorithm are based on the constrained NSGA-II algorithm. First, perform random initialization on $Population{\kern 1pt} {\kern 1pt} {\kern 1pt} {\kern 1pt} {\kern 1pt} 1$ and $Population{\kern 1pt} {\kern 1pt} {\kern 1pt} {\kern 1pt} 2$. During each iteration, the biparental sets Parent1 and Parent2 are selected from the two populations by a tournament mating selection strategy. The parents then generate an offspring population using simulated binary crossover operators and polynomial variations, respectively. After producing offspring, add the two offspring to $Population{\kern 1pt} {\kern 1pt} {\kern 1pt} {\kern 1pt} {\kern 1pt} 1$ and $Population{\kern 1pt} {\kern 1pt} {\kern 1pt} {\kern 1pt} 2$. $Population{\kern 1pt} {\kern 1pt} {\kern 1pt} {\kern 1pt} {\kern 1pt} 1$ is selected using constrained non-dominant sorting and shift-based crowding distance. $Population{\kern 1pt} {\kern 1pt} {\kern 1pt} {\kern 1pt} 2$ dynamically changes the selection of constraint information weights according to the proportion of feasible solutions and the number of iterations to realize the dynamic adjustment of constraints. In the last iteration, the solution in $Population{\kern 1pt} {\kern 1pt} {\kern 1pt} {\kern 1pt} 2$ can also be output as the final solution since the dynamic constraint selection strategy of $Population{\kern 1pt} {\kern 1pt} {\kern 1pt} {\kern 1pt} 2$ has fully considered the constraints. Therefore, the two populations are merged for the final environmental selection and returned as the final result output. The specific process of CDSMO algorithm is shown in Algorithm 2.

**Algorithm 2:**
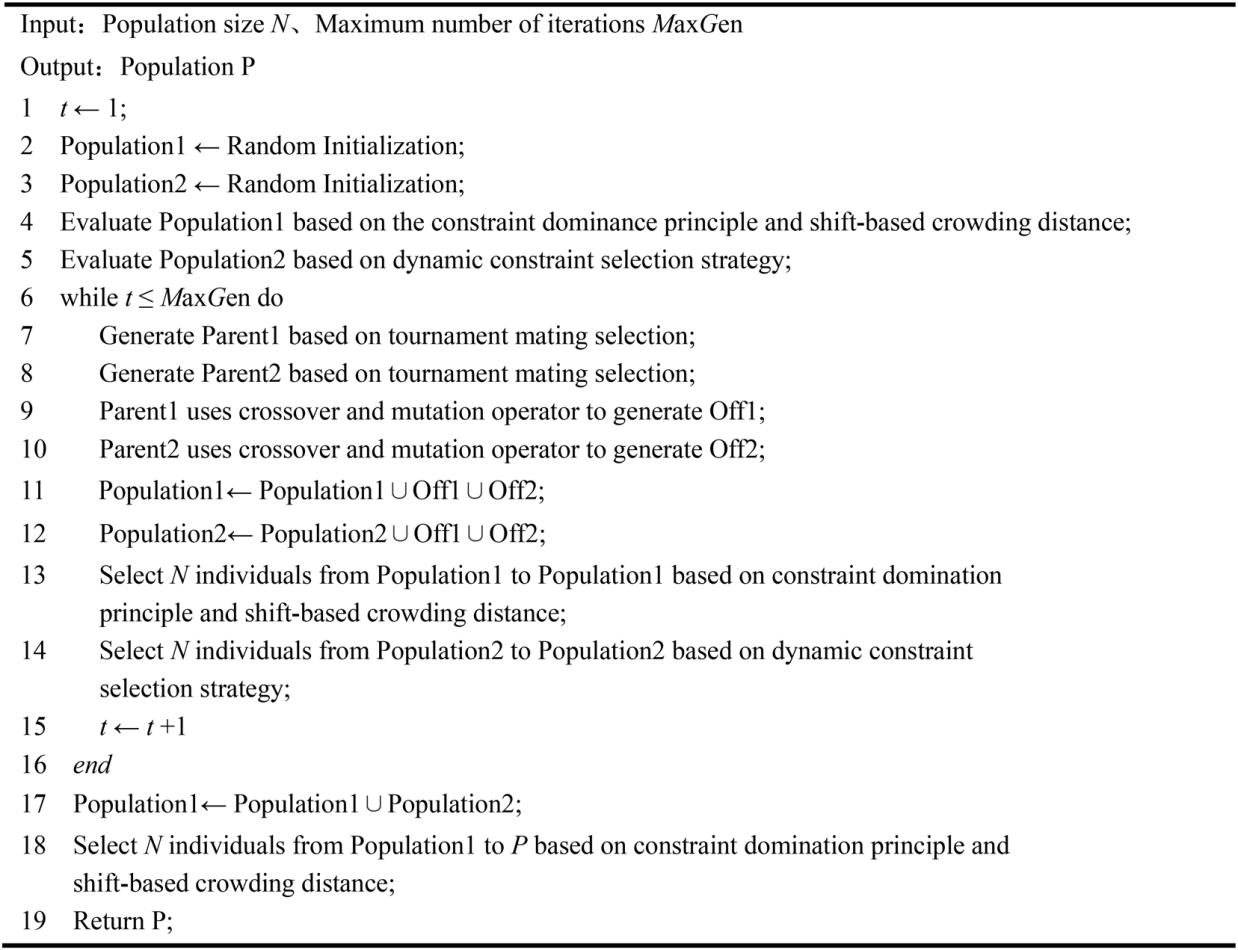
CDSMO Algorithm Flow

### Dynamic constraint selection strategy

**Fig. 2:**
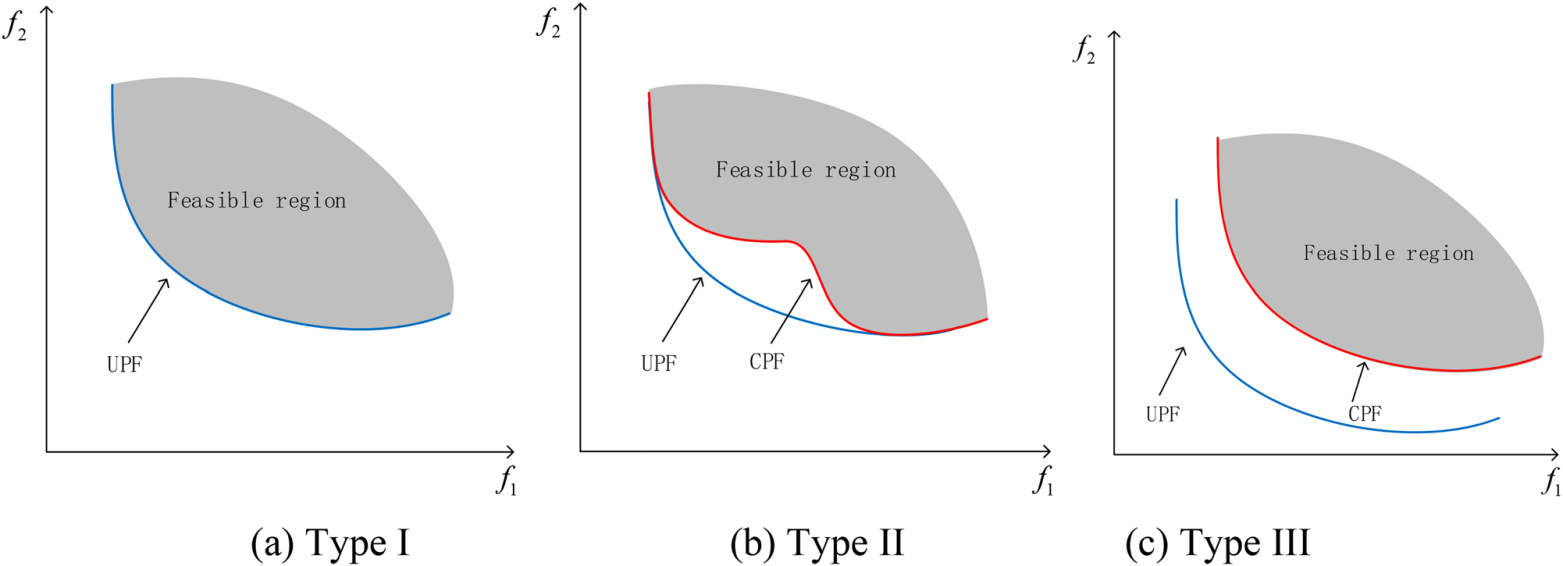
Classification of Constrained Multi objective Problems.

Based on the relationship between unconstrained Pareto front (UPF) and constrained Pareto front (CPF), the existing constrained multi-objective problems can be divided into three categories, as shown in Fig. 2. Type I: Completely overlap between CPF and UPF. Type II: Partial overlap between CPF and UPF. Type III: CPF and UPF completely do not overlap. A schematic of the three types is given in Fig. 2. The auxiliary population of existing co-evolutionary algorithms does not consider any constraint violations and can quickly converge to UPF, allowing the main population to also approach UPF^35,36^. Thus, these co-evolutionary algorithms perform well in Type I, and Type II is more complex than Type I. These algorithms can easily find the overlapping parts of CPF and UPF, and it is more difficult for the non-overlapping parts. For type III, the CPF and UPF do not overlap, and the CPF is completely composed of feasible region boundaries. The auxiliary population can help the main population to traverse the infeasible region to find find segmented feasible region and provide some useful information to the main population when passing through the CPF. Since the auxiliary population does not consider constraints, it always stays in this region after reaching the UPF. The offspring population is often generated near the initial population, so the auxiliary population will always have a certain distance from CPF, and the larger the distance, the worse the performance of the algorithm.

As shown in Algorithm 3, a dynamic constraint selection strategy is designed for assisting the population to solve the above problem. The strategy dynamically adjusts the impact of constraint information on environmental selection based on the number of iterations and the proportion of feasible solutions. Specifically, for each individual $x_{i}$ in the population, rank $Rp_{i}$ is obtained by first ordering based on Pareto dominance and crowding distance, and then rank $Rc_{i}$ is obtained by a second ordering based on CDP and crowding distance. $Rp_{i}$ is based on the objective function and ignores constraints. $Rc_{i}$ considers both objective function and constraints simultaneously. For both ranks, smaller values indicate better performance. The final rank of the population is calculated using Eq. (1).

21 $$R_{i} (t) = w(t,P_{fea} ) \cdot Rp_{i} + (1 - w(t,P_{fea} )) \cdot Rc_{i}$$

The weight $w$ is calculated based on the number of iterations $t$ and the proportion of feasible solutions $P_{fea}$ in the current population, and is calculated as follows:

22 $$w(t,P_{fea} ) = base \cdot (1 - \frac{t}{\max Gen})^{{\frac{1}{{P_{fea} + k}}}}$$

where, $base$ is the initial weight value, which is normally set to 0.9^35^ and $k$ is a smaller number, which is normally set to 0.001^36^.

**Algorithm 3:**
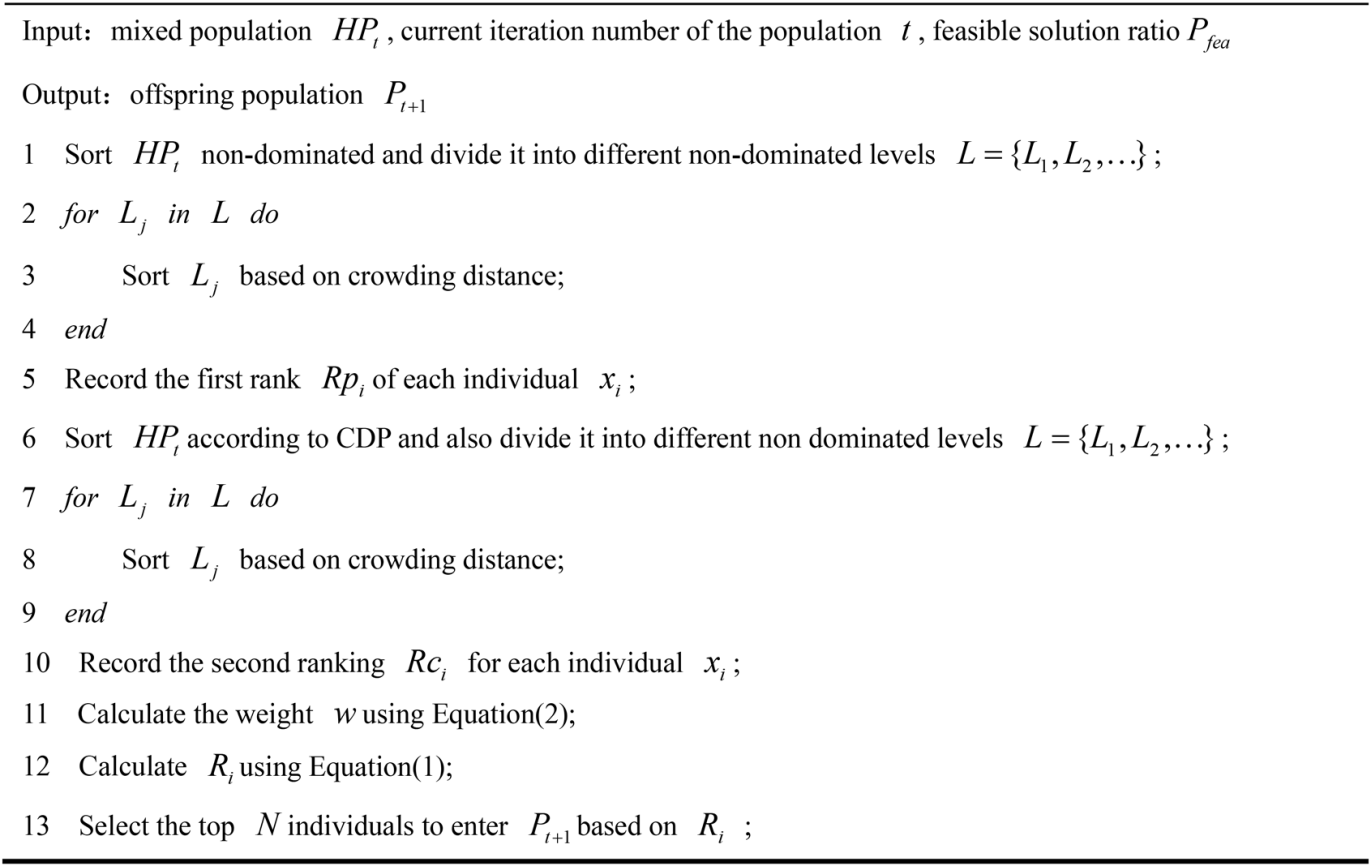
Dynamic constrain selection strategy

Throughout the iteration process, the overall trend of the weights $w$ is determined by the number of iterations $t$, and the weight decreases with the increase of $t$. With the continuous evolution of the population, selection strategies need to consider more constraint information, and constraints have become increasingly important. In the final evolutionary stage, the population will consider complete constrained information.

For a certain iteration, the proportion of feasible solutions for the previous generation of populations determines the weight of the current population. $P_{fea}$ is 0 at the initial stage, and $w$ is close to $base$, which is a large value. This ensures that infeasible solutions with good objective values are retained. The population can approach the feasible region from different directions with the help of these infeasible solutions, thus avoiding premature convergence. When the proportion of feasible solution $P_{fea}$ in the population is larger, $w$ is smaller, and the population considers more objectives. Reducing the weight of constraints is beneficial for maintaining population diversity, encouraging the population to explore more discontinuous feasible regions, and helping to search for Pareto optimal solutions located on the boundary of the feasible of feasible regions. When the proportion of feasible solutions $P_{fea}$ in the population is small, $w$ is larger, and the population is more biased towards constraint information, providing more selection pressure for infeasible solutions to converge to the feasible domain and obtaining more feasible solutions.

### Crowding distance calculation

In a multi-objective optimization algorithm based on the Pareto dominance relation, the proportion of non-dominant individuals in the population increases dramatically as the number of objectives increases, resulting in the invalidation of the Pareto dominance relation. In extreme cases, even all individuals in the population are non-dominant individuals. At this point, the diversity maintenance mechanism (individual density) plays a dominant role in the evolutionary selection process of the algorithm. Whereas the preference of the diversity maintenance mechanism for individuals in sparse regions (i.e., individuals with low similarity to other individuals) leads to final solutions that are widely distributed in the target space, but far from the expected Pareto frontier. For example, individuals are slightly better than others on some objectives, but significantly worse on at least one objective. Consider three non-dominant individuals with objective values of (0, 1100), (1, 0, 2), and (2, 1, 0). Among them, the first individual performs the worst in terms of convergence, but is a non-dominat solution in the algorithm based on the Pareto dominance relation (the first objective value is 0). This diversity maintenance mechanism seriously deteriorates the search performance of the algorithm. In mating selection, non-dominant individuals with poor convergence (e.g., the first individual in the above example) have a higher probability of being selected and generating offspring with poorer performance. In environmental selection, the prolonged presence of poorly convergent individuals can lead to the elimination of some well-convergent individuals due to population size constraints.

Shift-based density estimation (SDE) is used in the CDSMO algorithm for solving this problem^37^. Different from the traditional crowding distance that only considers the distribution of individuals in the population, SDE considers both individual distribution and convergence information, effectively balancing the convergence and diversity of solutions.

The traditional crowding distance is calculated based on the relative position of other individuals towards that individual. In SDE, the position of other individuals is adjusted to reflect the convergence of individuals in the population. When estimating the crowding distance of individual $a$, the SDE moves the individual position based on the convergent comparison between other individuals and $a$ on each objective. More specifically, if an individual $b$ performs better than $a$ on an objective, it will be adjusted to the same position as $a$, otherwise it will remain the same. The shifted individual $b^{\prime}$ is shown in Eq. (23).

23 $$b^{\prime}(j) = \ge \left\{ {\begin{array}{*{20}l} {a(j),} \hfill & {if\,\,b(j) < a(j)} \hfill \\ {b(j),} \hfill & {otherwise} \hfill \\ \end{array} } \right.,j \in (1,2, \ldots ,m)$$

where $a(j)$, $b(j)$, and $b^{\prime}(j)$,denote the j-th target value for individuals $a$,$b$, $b^{\prime}$, and $m$ denotes the number of targets.

**Fig. 3:**
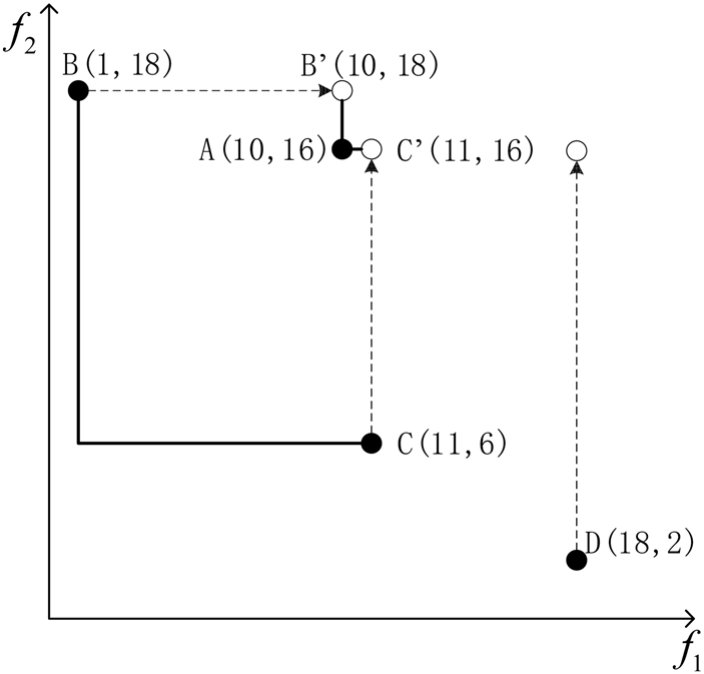
Example of shift-based density estimation.

**Fig. 4:**
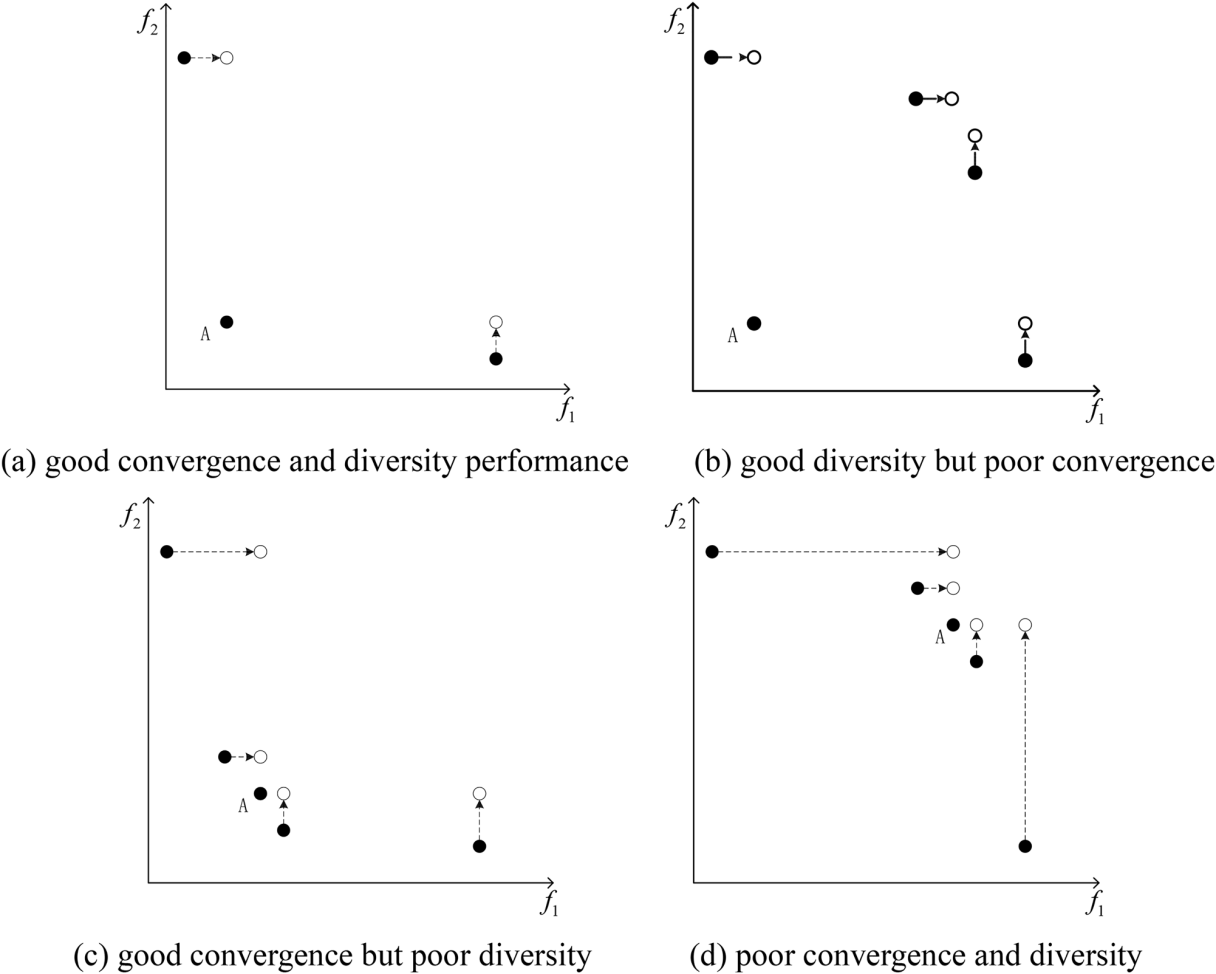
Crowding distance of individual A in different situations.

An example of SDE is given in Fig. 3, where A has a higher congestion after using SDE calculation. This is due to the existence of two individuals B and C which perform significantly better than A in terms of convergence, that is, slightly lower than A on one or some objectives but better than A on others. This means that individuals with no significant advantage in the population will have high density values in SDE. Figure 3 shows four typical distributions of individuals in a population: good convergence and diversity performance, good diversity but poor convergence, good convergence but poor diversity, and poor convergence and diversity, as shown in Figs. 4 (a), (b), (c), and (d), respectively. It can be observed that only individuals with both good convergence and diversity have lower crowding in SDE. Individuals with poor convergence or diversity have some close neighbors, and individuals with poor both have the highest crowding.

### Crossover and mutation

This paper studies the green vehicle routing problem with time window constraints considering capacity and time-varying factors. The solution sought is typically represented by a permutation of customers, where each vehicle’s travel route is separated by a distribution center. Therefore, the CDSMO algorithm uses sequence based crossover and mutation operators to generate offspring. The crossover operator uses the sequential crossover operator, and the mutation operator uses the exchange mutation operator.

The steps of the sequential crossover operator are as follows:

1. Randomly select two different individuals from the population as parents and generate a random number $r$. If $r$ is less than the crossover probability $P_{c}$, then the crossover operator operation is performed, otherwise the crossover operation is skipped directly.
2. The crossover operator randomly selects a route from the first and second parent individuals, respectively.
3. For the first parent individual, insert the randomly selected route of the second parent individual into the corresponding position. Then the missing customer points are inserted in the order of the first parent individual to generate a new child individual.

## Experiment and result analysis

### Experimental setup

Experimental environment: MATLAB 2021a, computer configuration Intel Core i7—3630QM 2.40 GHz, 8 GB RAM, executed in window 11 system.

Parameter setting: To select the optimal parameter configuration, this paper conducted preliminary experimental tuning to evaluate the impact of these parameters on the algorithm performance, and also referred to the existing literature. The final parameter settings are as follows:

To adapt to the problem model proposed in this paper, the whole working time is divided into three segments on all examples. The proportion of each segment in the total working interval is [0.1,0.8,0.1], the corresponding speed is [0.65,1,0.55], and the speed unit is km/min. The maximum load capacity $$Cap$$ of the distribution vehicle is 200 kg, the fixed cost $$c_{v}$$ of the vehicle is 200$, the maximum allowable early arrival or delay time $$md$$ for customers is 15 min, the unit distance travel cost $$c_{d}$$ of the vehicle is 5 $/kilometer, the unit delay time penalty cost $$c_{t}$$ is 20 $/min, and the sequential crossover probability is 0.9.

Datasets: This study selected two benchmark datasets, including the Solomon dataset and the Homerger dataset, and the same actual dataset used by Zhou and Wang^38^ and Castro Gutierrez et al.^39^, This instance contains a combination of five different time window configuration strategies (i.e.$tw0$,$tw1$,$tw2$,$tw3$ and $tw4$) and three vehicle load capacities (i.e.$d0$,$d1$ and $d2$).The vehicle load is calculated according to formula $C = d_{\max } + \delta /100(D - d_{\max } )$, and the three types of vehicle load are calculated when $\delta$ takes a value of $\{ 60205\}$, where $d_{\max }$ represents the maximum demand of customers and $D$ is the total demand of all customers. This dataset and configuration file can be downloaded from^40^.

These five time window configuration types are described as follows:

Type 1: All customers are available 24 h/7d, which means the entire 8 h (480 min).

Type 2: Consider three types of customers, namely early customers, noon customers, and late customers. The total time of 480 min is divided into three equal parts, which are 160 min time slots for each type of customer. Early customers will receive service within [0,160) minutes, noon customers will receive service within [160,320) minutes, and late customers will receive service within [320,480] minute intervals.

Type 3: Reduce the length of each time window by 30 min. Therefore, the time period for early customers is [0,130], the time period for mid-term customers is [175,305], and the time period for late customers is [350,480].

Type 4: Further shorten the length of each time window by 30 min. Therefore, the time interval for early customers is [0,100], the time interval for mid-term customers is [190,290], and the time period for late customers is [380,480].

Type 5: Customers can belong to any of the above time window configuration files.

$tw0$ only contains the first type of time window, $tw1$, $tw2$, and $tw3$ contain types 2, 3, and 4 respectively, $tw4$ includes all types of time windows, and the probability of each time window type appearing in each configuration file is the same.

Comparison algorithm: Since MOGVRPTW-TV is a new problem and there are no existing algorithms and results for direct comparison, this paper compares NSGAII^41^(a multi-objective optimization algorithm), CCMO^42^, cDPEA^43^ (a constrained multi-objective optimization algorithm) for the problem. The parameters of all algorithms are set as suggested in the original paper. All algorithms are executed under identical conditions, which means using the same starting and ending criteria, the same number of starting search points, the same dataset, and the same hardware for running the algorithm. To reduce the influence of randomness, all experiments were carried out 30 times.

### Results analysis of Solomon dataset

Tables 4, 5, and 6 give the comparison results of each constrained multi-objective optimization algorithm on the Solomon dataset when the customers are randomly distributed, aggregated distributed and mixed distributed, respectively. In the table, $$TC$$, $$TT$$, and $$NV$$ represent the total transportation cost (including carbon emission costs affected by load and speed), the total travel time, and number of vehicles, in units of $, min, and number of vehicles, respectively. In addition, all tables also provide the computation time of each algorithm in different instances, represented by $$T$$(unit: seconds).

**Table 4:** Comparison of different algorithms on the randomly distributed Solomon dataset.

Instance ID	NSGAII				CCMO				cDPEA				CDSMO			
	*TC*	*TT*	*NV*	*T*	*TC*	*TT*	*NV*	*T*	*TC*	*TT*	*NV*	*T*	*TC*	*TT*	*NV*	*T*
R20	4705	979	6	36	4686	911	5	32	4391	870	5	30	4214	869	5	28
R40	9431	1796	11	365	8686	1687	10	352	8887	1676	10	347	8265	1627	10	330
R60	14,566	2679	16	552	12,194	2321	14	513	12,745	2494	15	478	12,021	2298	13	432
R80	19,264	3487	20	875	16,833	3228	19	817	18,309	3312	19	768	16,311	3152	19	731
R100	23,845	3832	23	997	21,086	3701	23	966	22,412	3787	23	904	19,443	3684	22	892

**Table 5:** Comparison of different algorithms on the cluster distribution Solomon dataset.

Instance ID	NSGAII				CCMO				cDPEA				CDSMO			
	*TC*	*TT*	*NV*	*T*	*TC*	*TT*	*NV*	*T*	*TC*	*TT*	*NV*	*T*	*TC*	*TT*	*NV*	*T*
C20	3355	2011	2	89	4070	2313	3	74	2992	1970	2	66	2955	2011	2	47
C40	8057	4387	5	567	7952	4410	5	512	7961	4403	5	489	6836	4035	4	435
C60	12,361	6754	8	765	11,853	6516	7	698	12,216	6435	7	627	11,942	6675	7	577
C80	17,731	9018	10	995	18,850	9138	10	933	21,896	10,027	11	908	17,087	8977	10	891
C100	23,913	11,288	13	1202	24,206	11,967	13	1165	23,367	12,161	14	1054	21,640	10,948	12	993

**Table 6:** Comparison of different algorithms on the mixed distribution Solomon dataset.

Instance ID	NSGAII				CCMO				cDPEA				CDSMO			
	*TC*	*TT*	*NV*	*T*	*TC*	*TT*	*NV*	*T*	*TC*	*TT*	*NV*	*T*	*TC*	*TT*	*NV*	*T*
RC20	3634	730	4	24	3397	736	4	20	3374	729	4	19	3350	699	4	15
RC40	7101	1485	8	223	7290	1519	7	268	7282	1506	8	249	7047	1386	7	215
RC60	11,956	2271	12	384	11,580	2159	11	346	11,144	2094	12	319	10,703	2042	11	303
RC80	14,970	2995	16	624	14,916	3044	15	583	14,278	3017	15	545	14,214	2793	15	514
RC100	19,441	3745	20	768	19,264	3731	20	719	20,985	4098	20	684	17,876	3565	19	647

By testing a total of 15 instances of 20 to 100 customer types with different distributions on the Solomon benchmark dataset, it can be seen that: The CDSMO algorithm proposed in this paper shows better performance. On instance R100, when the CDSMO algorithm is used for VRP, the total transportation cost for completing distribution services is $19443, the total travel time is 3684 min, and the number of vehicles is 22. Compared to CCMO, which has the best total transportation cost among NSGA-II, CCMO, and cDPEA, the total transportation cost of completing the distribution service is reduced by about 7.8%, the total travel time is reduced by about 0.5%, and the number of vehicles used is reduced by 1. On instance C100, when the CDSMO algorithm is used for VRP, the total transportation cost for completing distribution services is $21640, the total travel time is 10948 min, and the number of vehicles used is 12. Compared to cDPEA, which has the best total transportation cost among NSGA-II, CCMO, and cDPEA, the total transportation cost of completing the distribution service is reduced by about 7.4%, the total travel time is reduced by about 9.9%, and the number of vehicles used is reduced by 2. On instance RC100, the total transportation cost for completing distribution services is $17,876, the total travel time is 3,565 min, and the number of vehicles used is 19. In this case, the total transportation cost of completing the distribution service is reduced by about 7.2%, the total travel time is reduced by about 4.4%, and the number of vehicles used is reduced by 1.

To better prove the efficiency of the algorithm proposed in this paper, this paper randomly selects instances of different sizes and types on the Solomon dataset and compares them with the multi-objective optimization algorithms MOEA/D^44^ and SPEA2^45^. The experimental comparison results are shown in Table 7.

**Table 7:** Comparison of different multi-objective optimization algorithm on Solomon dataset.

Instance ID	_MOEA/D_				_SPEA2_				CDSMO			
	*TC*	*TT*	*NV*	*T*	*TC*	*TT*	*NV*	*T*	*TC*	*TT*	*NV*	*T*
R40	8324	1709	11	338	8890	1947	13	430	8265	1627	10	330
R80	17,023	3201	20	762	19,205	3425	25	802	16,311	3152	19	731
C20	3014	2104	3	52	3320	2301	5	65	2955	2011	2	47
C80	17,986	9021	11	903	19,203	9248	15	945	17,087	8977	10	891
RC60	11,024	2102	12	312	12,035	2358	15	356	10,703	2042	11	303
RC100	18,205	3569	20	655	19,685	3689	22	690	17,876	3565	19	647

It can be seen from Table 7: the CDSMO algorithm proposed in this paper shows better performance. Taking the instance RC60 as an example, when the CDSMO algorithm is used, the total transportation cost for completing distribution services is $10,703, the total travel time is 2042 min, and the number of vehicles is 11. Compared to MOEA/D, which has the best total transportation cost among MOEA/D, and SPEA2, the total transportation cost of completing the distribution service is reduced by about 3.0%, the total travel time is reduced by about 3.9%, and the number of vehicles used is reduced by 1.

**Fig. 5:**
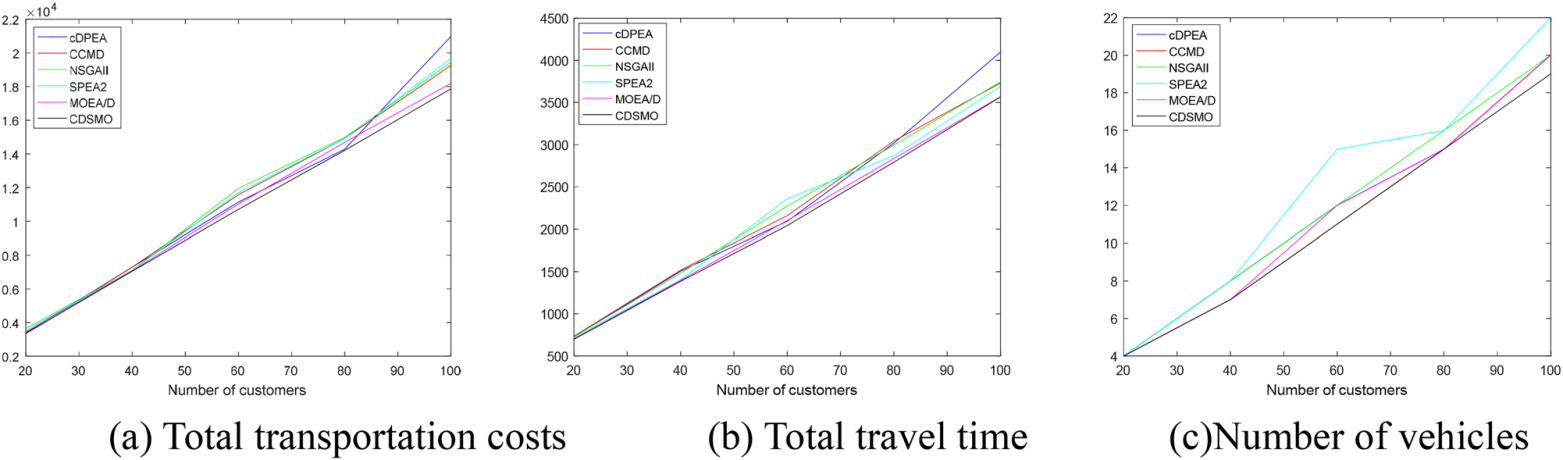
The trend of each target changing with the growth of customer size under different algorithms.

To show more intuitively the relationship between different customer sizes and target values, Fig. 5a–c show the trend of different algorithms for each target in the mixed distribution type. The horizontal coordinate is the customer size and the vertical coordinate is the target value.

From Fig. 5a–c, it can be seen that as the customer size increases, the target value steadily increases. The CDSMO algorithm grows slower and more steadily than the other algorithms. The CDSMO algorithm is able to take the optimal results under different sizes, proving the effectiveness of the proposed algorithm in solving different customer sizes.

To analyze the significance of the differences in the experimental results, the experimental results of all algorithms were T tested by randomly selecting examples of different sizes and types. The test results are shown in Table 8. It can be seen from Table 8: Based on the T-test results, both the total transportation cost and the total travel time are significantly different than each of the other methods.

**Table 8:** T-test results.

Instance ID	*TC*						*TT*					
	NSGAII	CCMO	cDPEA	MOEA/D	SPEA2	CDSMO	NSGAII	CCMO	cDPEA	MOEA/D	SPEA2	CDSMO
R40	− 8.25	− 5.98	− 3.25	− 1.16	− 1.20	–	− 6.68	− 5.98	− 5.65	− 5.13	− 5.20	–
R80	− 7.98	− 5.65	− 3.06	− 1.14	− 1.18	–	− 6.54	− 5.65	− 5.23	− 4.63	− 4.96	–
C20	− 9.25	− 6.32	− 3.87	− 2.35	− 2.04	–	− 7.32	− 6.03	− 5.96	− 5.22	− 5.29	–
C80	− 8.25	− 6.02	− 3.19	− 2.01	− 1.68	–	− 6.35	− 5.89	− 5.02	− 4.63	− 5.27	–
RC60	− 7.25	− 5.96	− 3.15	− 2.06	− 1.64	–	− 6.34	− 6.02	− 5.98	− 5.15	− 5.36	–
RC100	− 6.93	− 5.68	− 3.02	− 1.98	− 1.08	–	− 6.32	− 5.67	− 5.32	− 5.02	− 5.11	–

### Results analysis of Homberger dataset

To further validate the performance of the CDSMO algorithm proposed in this paper, eight sets of large-scale instances from 100 to 800 customers on the Homberger dataset are selected for validation. Table 9 shows the experimental results of all comparison algorithms on instances of different scales.

**Table 9:** Comparison of different algorithms on Homberger dataset.

Instance ID	NSGAII			CCMO			cDPEA			CDSMO		
	*TC*	*TT*	*NV*	*TC*	*TT*	*NV*	*TC*	*TT*	*NV*	*TC*	*TT*	*NV*
100	19,502	3801	20	19,328	3796	20	21,026	4125	20	17,993	3638	19
200	32,519	6978	31	32,423	6939	29	33,175	7011	32	32,092	6834	27
300	49,108	11,087	60	48,975	11,028	58	49,764	11,108	60	48,643	10,873	55
400	64,645	19,276	105	64,576	19,208	102	64,762	19,304	106	64,397	18,276	98
500	82,018	28,785	137	81,923	28,732	132	82,133	28,906	138	81,863	27,998	126
600	98,723	40,086	169	98,654	40,013	165	98,837	40,109	171	98,386	39,823	154
700	118,012	50,802	206	117,986	50,762	202	118,108	50,902	208	117,623	50,177	188
800	131,316	63,304	251	131,276	63,254	247	131,387	63,407	254	131,092	63,082	236

By testing 8 sets of examples on the Homerger dataset, it can be seen that the CDSMO algorithm proposed in this paper shows better performance. As can be seen from Table 9, the values of each objective function increase multiplicatively as the number of customers increases. When the number of customers exceeds 500, the growth of each objective function tends to stabilize. Taking 500 customers as an example, when CDSMO algorithm is used for VRP, the total transportation cost for completing distribution services is $81,863, the total travel time is 27,998 min, and the number of vehicles used is 126. Compared to CCMO, which has the best total transportation cost among NSGA-II, CCMO, and cDPEA, the total transportation cost of completing the distribution service is reduced by about 0.07%, the total travel time is reduced by about 2.6%, and the number of vehicles used is reduced by 6.

Similarly, to better prove the efficiency of the algorithm proposed in this paper, this paper randomly selects instances of different sizes and types on the Homberger dataset and compares them with the multi-objective optimization algorithms MOEA/D^44^ and SPEA2^45^. The experimental comparison results are shown in Table 10. And the T-test results are shown in Table 11.

**Table 10:** Comparison of different multi-objective optimization algorithm on Homberger dataset.

Instance ID	_MOEA/D_			_SPEA2_			CDSMO		
	*TC*	*TT*	*NV*	*TC*	*TT*	*NV*	*TC*	*TT*	*NV*
200	33,025	6903	29	35,602	7021	31	32,092	6834	27
400	65,021	19,025	99	66,307	19,987	102	64,397	18,276	98
600	99,325	40,213	158	102,654	41,369	167	98,386	39,823	154
800	136,982	64,503	246	153,698	66,308	268	131,092	63,082	236

**Table 11:** T-test results.

Instance ID	*TC*						*TT*					
	NSGAII	CCMO	cDPEA	MOEA/D	SPEA2	CDSMO	NSGAII	CCMO	cDPEA	MOEA/D	SPEA2	CDSMO
200	− 2.08	− 1.96	− 1.03	− 0.85	− 0.96	–	− 4.17	− 3.68	− 3.56	− 3.18	− 3.21	–
400	− 2.01	− 1.89	− 1.01	− 0.84	− 0.92	–	− 3.98	− 3.38	− 3.42	− 3.11	− 3.16	–
600	− 1.97	− 1.82	− 0.96	− 0.83	− 0.89	–	− 3.69	− 3.29	− 3.25	− 3.02	− 3.08	–
800	− 1.93	− 1.81	− 0..96	− 0.81	− 0.85	–	− 3.58	− 3.22	− 3.17	− 3.01	− 2.99	–

It can be seen from Table 10: the CDSMO algorithm proposed in this paper shows better performance. Taking the instance 600 as an example, when the CDSMO algorithm is used, the total transportation cost for completing distribution services is $98,386, the total travel time is 39,823 min, and the number of vehicles is 154. Compared to MOEA/D, which has the best total transportation cost among MOEA/D, and SPEA2, the total transportation cost of completing the distribution service is reduced by about 0.95%, the total travel time is reduced by about 0.97%, and the number of vehicles used is reduced by 4. Based on the T-test results in Table 11, both the total transportation cost and the total travel time are significantly different than each of the other methods.

**Fig. 6:**
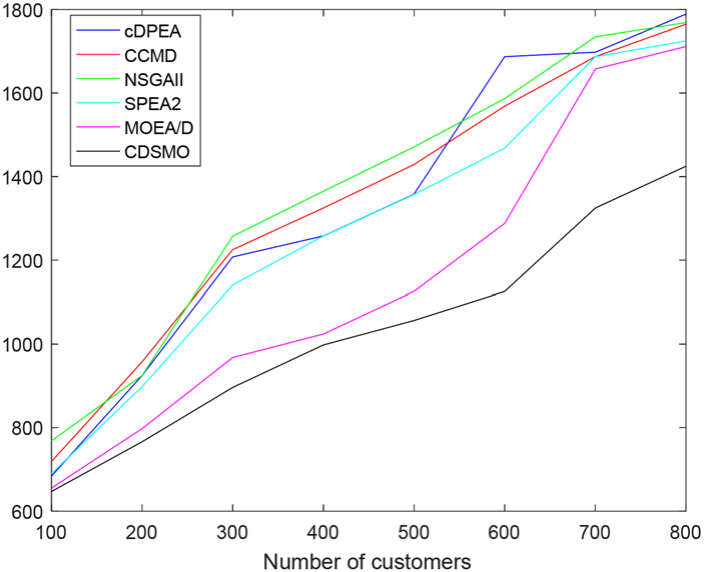
Running time trend chart of different algorithms.

Since the maximum size of the Homberger dataset is set to 800 customers, for scalability on a very large scale (such as 1000 + customers), Fig. 6 shows the actual running time trends of different algorithms. It can be seen from Fig. 6 that with the expansion of customer scale, the running time of the algorithm increases gradually, but the running time of the algorithm proposed in this paper is significantly lower than that of other algorithms, indicating the efficiency of the algorithm proposed in this paper.

### Results analysis of actual MOVRPTW dataset

The experiment is carried out on a real MOVRPTW dataset. The data used in this paper include 50 customers, where the vehicle loads and the time window settings can be combined into 15 instances. The instance ID consists of a vehicle load and time window, e.g., $d0tw0$ and $d0$ represent the 0-th load, and $tw0$ represents the 0-th window type. As the number increases, both the load and the window size decrease. Table 12 gives the experimental results of different comparison algorithms on the actual MOVRPTW dataset.

**Table 12:** Results of different algorithms on actual MOVRPTW instances.

Instance ID	NSGAII			CCMO			cDPEA			CDSMO		
	*TC*	*TT*	*NV*	*TC*	*TT*	*NV*	*TC*	*TT*	*NV*	*TC*	*TT*	*NV*
*d*0*tw*0	6636	1820	7	7066	1952	6	6772	1890	6	6215	1840	6
*d*0*tw*1	10,754	3320	8	9284	3175	7	9188	3147	7	8909	3135	7
*d*0*tw*2	10,681	3429	7	10,775	3405	7	10,567	3440	7	9584	3391	7
*d*0*tw*3	12,199	3999	8	11,611	4047	8	12,276	4001	8	10,823	3889	8
*d*0*tw*4	10,721	2594	7	9541	2877	7	9667	2684	7	8705	2616	7
*d*0*tw*0	7125	1917	7	6992	1863	7	7096	1948	6	6486	1861	6
*d*0*tw*1	10,916	3185	7	10,138	3236	7	10,127	3125	7	9124	3131	7
*d*0*tw*2	11,313	3488	7	10,550	3428	7	10,870	3458	7	9616	3392	7
*d*0*tw*3	11,933	3994	8	11,572	4011	8	10,954	3841	8	10,933	3896	8
*d*0*tw*4	9619	2586	7	9096	2784	7	8957	2713	7	8684	2528	7
*d*0*tw*0	12,234	2363	15	12,075	2340	15	12,543	2384	15	11,557	2273	15
*d*0*tw*1	17,055	5550	15	17,626	5627	15	15,804	5426	15	15,822	5323	15
*d*0*tw*2	17,937	5801	15	17,273	5880	15	16,696	5972	15	16,309	5752	15
*d*0*tw*3	18,072	6132	15	17,817	6379	15	17,220	6338	15	16,295	6206	15
*d*0*tw*4	16,956	4705	15	15,141	4680	15	15,369	4982	15	14,292	4486	15

As can be seen from Table 8, the CDSMO algorithm achieves better objective values on most of the instances. For $$d0tw0$$ with the maximum load and the most relaxed time window, the total transportation cost of the CDSMO algorithm is $6215, the total travel time is 1840 min, and the number of vehicles used is 6. Compared to the NSGA-II algorithm, which has the best total transportation cost among the remaining algorithms, the total transportation cost is reduced by about 6.3%, the total travel time is increased by about 1.1%, and the number of vehicles used is reduced by 1. For $$d0tw0$$, the capacity and time window are small constraints on the solution of the problem, thus causing the CDSMO algorithm returns to the normal level of NSGA-II, and failing to reflect the advantages of CDSMO in dominating the solution of NSGA-II. For $$d2tw4$$, there are complex constraints due to the extremely small load and the mix of multiple time window. The total transportation cost of the CDSMO algorithm is $14,292, the total travel time is 4486 min, and the number of vehicles used is 15. Compared with the CCMO algorithm, which has the best total transportation cost among other algorithms, the total transportation cost is reduced by about 5.6% and the total travel time is reduced by about 4.1%. The results show the effectiveness of the CDSMO algorithm to solve complex constrained multi-objective optimization problems.

Similarly, to better prove the efficiency of the algorithm proposed in this paper, this paper randomly selects instances of different sizes and types on actual MOVRPTW instances and compares them with the multi-objective optimization algorithms MOEA/D^44^ and SPEA2^45^. The experimental comparison results are shown in Table 13. And the T-test results are shown in Table 14.

**Table 13:** Comparison of different multi-objective optimization algorithm on actual MOVRPTW instances.

Instance ID	_MOEA/D_			_SPEA2_			CDSMO		
	*TC*	*TT*	*NV*	*TC*	*TT*	*NV*	*TC*	*TT*	*NV*
*d*0*tw*0	6405	1968	7	6508	2035	8	6215	1840	6
*d*0*tw*2	9756	3502	8	9936	3658	9	9584	3391	7
*d*0*tw*4	8896	2712	8	9032	2898	9	8705	2616	7
*d*0*tw*1	9325	3258	8	9458	3369	9	9124	3131	7
*d*0*tw*3	11,102	3998	9	12,368	4102	10	10,933	3896	8
*d*0*tw*2	16,698	5896	16	17,035	6032	16	16,309	5752	15
*d*0*tw*4	14,698	4569	16	15,362	4896	17	14,292	4486	15

**Table 14:** T-test results.

Instance ID	*TC*						*TT*					
	NSGAII	CCMO	cDPEA	MOEA/D	SPEA2	CDSMO	NSGAII	CCMO	cDPEA	MOEA/D	SPEA2	CDSMO
*d*0*tw*0	− 0.75	− 0.82	− 0.98	− 0.56	− 0.68	–	− 1.12	− 1.23	− 1.35	− 1.01	− 1.06	–
*d*0*tw*2	− 0.89	− 0.94	− 1.01	− 0.69	− 0.78	–	− 1.21	− 1.24	− 1.30	− 1.09	− 1.12	–
*d*0*tw*4	− 0.85	− 0.92	− 0.98	− 0.71	− 0.79	–	− 1.18	− 1.21	− 1.26	− 1.02	− 1.10	–
*d*0*tw*1	− 0.79	− 0.84	− 0.92	− 0.65	− 0.72	–	− 1.22	− 1.24	− 1.25	− 1.03	− 1.11	–
*d*0*tw*3	− 0.78	− 0.86	− 0.89	− 0.63	− 0.66	–	− 1.16	− 1.21	− 1.23	− 1.10	− 1.15	–
*d*0*tw*2	− 0.93	− 0.96	− 1.01	− 0.72	− 0.78	–	− 1.16	− 1.19	− 1.25	− 1.08	− 1.13	–
*d*0*tw*4	− 0.88	− 0.92	− 0.93	− 0.59	− 0.68	–	− 1.19	− 1.22	− 1.26	− 1.06	− 1.09	–

It can be seen from Table 10: the CDSMO algorithm proposed in this paper shows better performance. Taking the instance $$d0tw4$$ as an example, when the CDSMO algorithm is used, the total transportation cost for completing distribution services is $8705, the total travel time is 2616 min, and the number of vehicles is 7. Compared to MOEA/D, which has the best total transportation cost among MOEA/D, and SPEA2, the total transportation cost of completing the distribution service is reduced by about 2.19%, the total travel time is reduced by about 3.67%, and the number of vehicles used is reduced by 1. Based on the T-test results in Table 11, both the total transportation cost and the total travel time are significantly different than each of the other methods.

### Complexity analysis of the CDSMO algorithm

The time complexity of the CDSMO algorithm is mainly determined by the adopted NSGA-II for the two populations and the designed dynamic constraint selection strategy. Assume that M, N, and D are the number of targets, population size, and decision vector dimensions, respectively. The worst-case time complexity of mating selection, genetic operators, and environmental selection for the main population are$O(N)$,$O(ND)$, and $O(MN^{2} )$, respectively. The auxiliary population adopts a dynamic constrained selection strategy in environmental selection, calculated based on Pareto non-dominated ordering and constrained dominance principle, resulting in a time complexity of $O(2MN^{2} )$. Thus the time complexity of the mating selection, genetic operator, and environment selection of the CDSMO algorithm are $2 \times O(N/2) = O(N)$, $2 \times O([N/2]D) = O(ND)$ , and $O(MN^{2} ) + O(2MN^{2} ) = O(MN^{2} )$, respectively. Therefore, the proposed CDSMO algorithm has the same worst-case time complexity as the NSGA-II algorithm, but it is actually slower than the NSGA-II algorithm because each iteration executes two selection strategies. The space complexity of the algorithm depends on the population size and the calculation of the crowding distance matrix. The space complexity of the population is $O(N^{2} + ND)$.

## Conclusion

In this paper, the green vehicle routing optimization problem with time window constraints is considered. To solve the problems of logistics and distribution with a single solution objective and real-time speed changes during vehicle travel, a multi-objective green vehicle routing model with time window constraints in a time-varying environment is constructed, and a constrained multi-objective evolutionary algorithm based on a co-evolutionary framework is proposed. First, based on the constraints in vehicle routing planning, the solution of the three objectives of transportation cost, total travel time, and number of vehicles is considered. Secondly, based on the idea of simple task assisting complex task solution in multi-task optimization, a constrained multi-objective evolutionary algorithm based on co-evolutionary framework is designed for solution. The algorithm considers both individual distribution and the convergence information of the shift crowding distance calculation computed to balance the convergence and diversity of solutions. Then, a dynamic constraint selection strategy is designed for simple task implementation. Finally, the efficiency of the proposed algorithm is tested on standard datasets and real cases, and the experimental results show the efficiency of the proposed algorithm.

In future research, it is necessary to further combine real-world traffic data to improve the time-varying speed model and simulate dynamic traffic conditions to verify the robustness of the algorithm. Meanwhile, considering the possibility of noise in dynamic data, it is necessary to preprocess it to improve reliability. In addition, random or dynamic elements such as demand fluctuations and traffic interruptions are introduced to enhance the adaptability of the model, and practical validation is conducted through large-scale case studies with industry partners to improve the engineering applicability of the framework.

## Data Availability

The datasets used and/or analysed during the current study available from the corresponding author on reasonable request.
